# Supplementary material for: Subsequent memory effect in the inferior frontal gyrus revealed by fNIRS
Source: PLoS One. 2026 May 26;21(5):e0349349. doi: 10.1371/journal.pone.0349349 (PMC13210360; doi:10.1371/journal.pone.0349349)
Supplement: Attachment — Submitted filename: response_to_reviewers.pdf [file pone.0349349.s003.pdf]

TILBURG UNIVERSITY

School of Humanities and Digital Sciences

Comment:
In the Discussion and Conclusion section, the authors directly infer that the left IFG participates in semantic processing and contributes to the early stages of memory formation. However, the present study neither directly measured semantic processing nor provided an operational definition of “early stages.” Consequently, this conclusion lacks direct empirical support. While prior literature may aid in interpreting the observed results, it should not be used to draw definitive conclusions. The relevant claims should be revised to prevent overinterpretation.

We agree with the reviewer that we stated this point too strongly. We adapted this part of the discussion section to the following to soften this claim (see line 245 onward):

Date
April 10, 2026

E-mail
s.h.p.collin@tilburguniversity.edu

“The results suggest that IFG, particularly the left IFG, plays a key role in successful memory formation, as indicated by neural differences between later remembered and forgotten events. In particular, at the peak of hemodynamic activation, remembered events elicit higher activity compared to forgotten events. This pattern is consistent with the possibility that the region is involved in the transformation of sensory information into internal representations (Paller et al., 2002) in the PFC, which may later be consolidated into a longer lasting trace through interactions with the MTL (Simons et al., 2003). This interpretation is in line with earlier research suggesting that the IFG is involved in semantic processing (Poldrack et al., 1999). In particular, the IFG has been proposed to contribute during stages of encoding, where it may coordinate semantic representations distributed across the cortex (Gabrieli et al., 1996).”
